# From the Sensor to the Cloud: Intelligence Partitioning for Smart Camera Applications

**DOI:** 10.3390/s19235162

**Published:** 2019-11-25

**Authors:** Irida Shallari, Mattias O’Nils

**Affiliations:** Department of Electronics Design, Mid Sweden University, Holmgatan 10, 851 70 Sundsvall, Sweden; mattias.onils@miun.se

**Keywords:** intelligence partitioning, smart camera, WVSN, IoT, in-sensor processing, fog, cloud, energy-efficiency

## Abstract

The Internet of Things has grown quickly in the last few years, with a variety of sensing, processing and storage devices interconnected, resulting in high data traffic. While some sensors such as temperature, or humidity sensors produce a few bits of data periodically, imaging sensors output data in the range of megabytes every second. This raises a complexity for battery operated smart cameras, as they would be required to perform intensive image processing operations on large volumes of data, within energy consumption constraints. By using intelligence partitioning we analyse the effects of different partitioning scenarios for the processing tasks between the smart camera node, the fog computing layer and cloud computing, in the node energy consumption as well as the real time performance of the WVSN (Wireless Vision Sensor Node). The results obtained show that traditional design space exploration approaches are inefficient for WVSN, while intelligence partitioning enhances the energy consumption performance of the smart camera node and meets the timing constraints.

## 1. Introduction

This is the era of information and technology, where data from a variety of sensors and devices are merged into products and services for the end user [1,2,3]. The advancement in embedded electronics providing low cost devices with small form factor and low power consumption enables the design of smart sensors with in-sensor processing capabilities [4]. In the meantime, IoT applications have expanded with scenarios relying on sensor deployment in both indoor and outdoor environments. Hence, designing smart devices assuming the presence of a power plug would restrict their applicability. To facilitate the deployment we need battery-operated smart sensors capable of performing their tasks within the energy consumption constraint.

An alternative to the constrained resources of in-sensor computing is cloud computing [5]. It relies on a subscription-based use of cloud instances with well defined specifications regarding computational capabilities and network traffic. Depending on the scope of the application, the cloud can be used as an extensive storage unit for statistical analysis of large volumes of data. It can also become part of the processing in the WSN providing real-time support for the sensor and the end user. Our focus lies on the latter, considering the cloud as a computational entity in an IoT application, where the sensor can offload processing tasks and data via the Internet. The inclusion of Internet communication in an IoT environment raises insecurities regarding achieving a satisfactory quality of service (especially for hard real-time applications) due to significant variations in communication latency influenced by congestion, bandwidth, or distance.

Visual inspection has a prominent role in our daily activities. Similarly, VSNs play a significant role in the Internet of Things perspective, for example in environmental monitoring and smart cities. Smart cameras are sensor nodes that contain the imaging sensor, the processing device and a transceiver in the camera node. If we consider the computational requirements of a smart camera node, they vary depending on the imaging sensor. However, a common feature is the large volume of data produced per second, which can be in the range of kilobytes per second up to several gigabytes per second. This introduces a twofold problem, where on the one hand there is the significant energy consumption caused by in-node processing of a large volume of data. On the other hand, if we consider continuously streaming the data from the camera node to the cloud, this would result in high communication latency, which can affect the quality of service.

Considering the large volume of data produced from a WVSN, the constrained resources of a battery-operated smart camera and the latency for data transfer over the Internet raises the question of where the optimal place to allocate the image processing tasks would be. Current architectures focus on either of the two following options: all the processing is performed in the smart camera, and sporadic data is sent to the cloud for statistical analysis; or, all the data is continuously streamed to the cloud where all the processing is done. The first option could have a significant impact on the battery life, while the latter could affect the quality of service due to latency, and significantly increasing the Internet traffic for large-scale deployment. The distribution of the computational workload between the two processing elements can be an option, as well as the introduction of an intermediate processing layer such as fog computing [6]. The location of the fog closer to the sensor relieves the latency complexity, while also overcoming the energy consumption constraint present in the smart camera.

In this paper, we introduce intelligence partitioning as an approach towards energy efficient smart sensor nodes in an IoT environment. The main contributions of this work are:

- Analysis of the inter-effects that three processing entities and three classes of communication technologies have in the IoT architecture.
- Analysis of the trade-off between processing and communication workload in the smart sensor node.
- Defining energy efficient regions on the three implementation cases considered, based on the hierarchy of constraints.

The remainder of the paper is organised as follows. Section 2 provides an overview of the methods presented by state-of-the-art research for node energy-efficiency in IoT related scenarios such as mobile computing and battery-operated cameras. Section 3 introduces the methods used in our analysis. Section 4 and Section 5 present the results and discussion on the effects of intelligence partitioning in the node energy consumption and real-time performance.

## 2. Related Work

Energy efficiency in battery-operated devices has been an issue that has interested researchers for many years. Several approaches were investigated, from which we can define three major groups. The first group focuses on the hardware architecture, where techniques such as clock gating or power gating can be used to reduce the energy consumption. If we simply consider a design change where we reduce the clock frequency by 50%, it would reduce the power consumption by 87.5%, while doubling the execution time. Hence, the overall energy consumption would be reduced to a quarter of the initial energy consumption. Another approach considered is the use of sleep and stand-by modes when the device is inactive. Despite the effectiveness on energy consumption reduction of these two approaches, their implementation in devices that are generally active and with real-time performance constraints would affect the quality of service due to either the lower clock frequency, or due to delays in wake-up time. The third approach consists on migrating the data to remote processing elements to perform a part or all the processing tasks. This paper focuses on the latter approach, investigating the effects that the distribution of image processing tasks has in the energy consumption and the overall processing and communication delay.

In the myriad of systems constituting the IoT, WVSN systems have a higher complexity than most smart sensor systems, due to the extensive amount of data to be processed and transferred. The WVSN approach for energy efficiency consists mainly of either processing everything in the smart camera, or the frames captured being continuously streamed to host processing units. For cases where in-sensor processing is used, energy efficiency is obtained through hardware and software optimised implementation of specific algorithms as in the case of DreamCam [7] with the feature extraction hardware. Other smart cameras, such as SENTIOF-CAM [8] and MeshEye [9], rely on the energy savings resulting from duty-cycling with predefined constraints, while [10,11] use external elements such as passive infrared detectors to switch between the sleep and wake-up modes. In contrast to in-node processing, some applications process all the data in a remote processing unit that can be a nearby server or a public cloud service [12]. Considering the high data volume to be transferred, which would also impact the node energy consumption, their focus is on implementing energy efficient data reduction methods. In addition to the frame-based image processing considered so far, other smart camera architectures have analysed the energy efficiency of the node caused by video encoding and cloud computing [13].

Mobile cloud computing is an environment similar to WVSN due to the hard constraints in computational resources and energy consumption. In the analysis of their energy-efficiency problem, the use of intermediate processing layers between the mobile device and the cloud, usually referred to as cloudlets, was included. The following papers [14,15,16,17,18], analyse the distribution of the processing tasks between the computational layers based on energy constraints, latency constraints, or a combination of the two. Their results suggest that the presence of cloudlets improves the energy-efficiency and latency of the mobile device. However, we should also take into consideration the difference in the constraints of real-time performance among WVSN and mobile computing, where the former requires about one order of magnitude shorter latency intervals, resulting in harder constraints than the latter.

The introduction of several processing layers, such as fog and cloud computing, requires a paradigm shift regarding design space exploration. Traditional methods focus more on total allocation of the processing tasks in only one computational entity, where all optimisation efforts are also concentrated. In our previous work we instead investigated the inter-effects of communication and processing in scenarios where the processing is partitioned between the smart camera node and a remote processing unit. We named this intelligence partitioning, and the results showed that the node energy efficiency is achieved for configurations where the processing tasks are partitioned between the node and the remote processing unit, instead of fully allocating them in either processing component. Considering the energy efficiency of the smart camera node achieved by intelligence partitioning, we decided to investigate the latency and possible applicability of such a system in the real world, considering fog and cloud processing with their incurring delays.

## 3. Methodology

IoT applications rely on sensor data and a set of computational tasks to be executed to achieve the scope of the application. Through the years the set of applications together with the computational entities have evolved, where each of these entities was analysed separately for implementation cases. To adapt the design space exploration for these architectural requirements, we investigate in the inter-effects that the computational entities and communication technologies have on the overall performance of the system. The term intelligence partitioning refers to the distribution of computational tasks among a number of computational entities, using several communication technologies, while focusing on the node energy consumption and real-time performance of the system.

**Figure 1:**
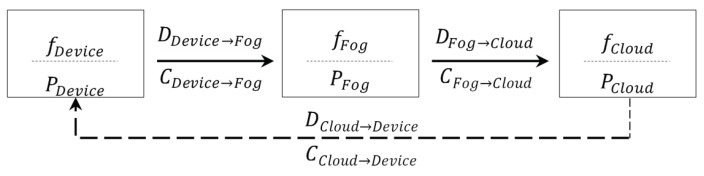
Schematic representation of intelligence partitioning between the node, fog and cloud computing layers.

The aim of this paper is to analyse aspects related to smart camera node energy consumption and the latency of a WVSN system consisting of node, fog and cloud computing. For the in-sensor processing, the smart camera supports both embedded hardware and software implementation of the image processing tasks. We base the analysis on the assumption that the allocation of any of the tasks is not restricted to a specific processing element. In addition, considering the processing flow in an image processing pipeline, where the successor task is fully dependant on the predecessor task, we consider only forward processing allocation starting from the smart camera node and moving towards the cloud, omitting the communication from the cloud to the smart camera node. This is represented in Figure 1 and Equation (1), where $f_{Node}$, $f_{Fog}$ and $f_{Cloud}$ represent the computational entities in the node, fog, and cloud, respectively, while *D* represents the data to be transferred between the computational entities.

(1) $$ℑ\left(F\right)=\{\begin{array}{c} \left\{f_{Node},f_{Fog},f_{Cloud}\right\}\\ D_{Node\to Fog},D_{Fog\to Cloud},D_{Node\to Cloud}, \end{array}$$

From the set of *N* image processing tasks to be executed in the WVSN, we denote by $S_{hw}$ and $S_{sw}$ the tasks allocated in embedded hardware and software in the smart camera, respectively. *F* represents the tasks allocated in the fog node, and *C* the set of tasks in the cloud instance. The energy consumption in the smart camera node can be formulated as:

(2) $$E_{node}=\sum_{i\in S_{hw}}^{}t_{i}\times P_{hw}+\sum_{j\in S_{sw}}^{}t_{j}\times P_{sw}+t_{wl}\times P_{wl}$$

$t_{i}$, $t_{j}$ and $t_{wl}$ represent, respectively, the execution time of the tasks implemented in embedded hardware, software and the time to transfer the data resulting from the smart camera processing (the node to fog communication time). Another element of interest in our analysis is the latency in the WVSN from the moment when a frame is captured until the end of the image processing pipeline. This can be formulated as the sum of the processing latency in each computational entity ($t_{f}$ and $t_{c}$ is the latency of a task implemented in the fog and cloud), to which is added the latency invoked by data transfers between the computational layers ($t_{wl}$ represents the latency for the node to fog communication, and $t_{I}$ is the latency for the fog to cloud communication via the Internet).

(3) $$L=\sum_{i\in S_{hw}}^{}t_{i}+\sum_{j\in S_{sw}}^{}t_{j}+t_{wl}+\sum_{f\in F}^{}t_{f}+t_{I}+\sum_{c\in C}^{}t_{c}$$

### 3.1. Processing Setup

The architecture of smart cameras consists of an imaging sensor, the processing element and a transceiver embedded in the same node. In Section 3.3 we introduce in more detail the people counting scenario used in our analysis. Regarding its implementation in the WVSN, the in-node processing element relies on a TE0726-03M Raspberry Pi that includes the Xilinx Zynq-7010 FPGA in its System on Chip (SoC) module. Due to constrained resources in the FPGA, only background modelling, segmentation and morphology were implemented in the programmable logic. However, all the image processing tasks were implemented in the processing system of the board, in the dual core ARM Cortex A9 processor. The estimation of the processing energy consumption in the smart camera node is calculated based on the power estimator tool provided by Xilinx [19].

In our WVSN architecture, in addition to the smart camera node, we considered the presence of fog and cloud computing elements, where the fog layer is allocated in the communication gateway and relies on a Raspberry Pi model 3B+. Regarding cloud computing, it is a widely used term that does not always refer to the same set of characteristics. Hence, in some publications the identifiers private and public are used to make a distinction between them. A private cloud represents a cloud instance with a location defined by the owner, with less computational resources than a public cloud, and generally it is connected through a wireless network [17]. A public cloud, however, is a cloud instance owned by a third party, located at specific points in the world and connected to the IoT system through an Internet connection. As the focus of this paper is on public clouds only, by cloud computing we will refer to this type of instance. This definition is of great importance for the following analysis in processing and communication latency of the image processing tasks. In the cloud computing layer we used a small instance from the Amazon Elastic Compute Cloud (EC2), with two virtual CPUs, 1 GB memory and a network performance of up to 5 Gigabit. For both the fog and cloud computing layers, the implementation of the image processing tasks for the people counting scenario was done using the OpenCV library.

### 3.2. Communication Setup

In the analysis of intelligence partitioning, the communication component has a major importance because of its influence in both the node energy consumption and the real-time performance of the WVSN. Regardless, WVSN architectures introduced in the state-of-the-art research focus on a specific communication technology chosen a priori [8]. In the meantime, the evolution in IoT applications has affected communication technologies resulting in the introduction of standards such as IEEE 802.15.4, NB-IoT and LoRa. Their aim is to support smart sensors with low energy consumption communication, at the cost of lower data rates and longer duty cycles. Considering only such technologies would limit our analysis to cases where all the processing is performed locally in the node, and sporadic data is sent to the fog or cloud. For this reason, we have considered three classes of communication technologies as shown in Table 1.

The estimation of the communication energy consumption and latency for the technologies in Table 1 is based on the model of Krug et al. [30], with point-to-point communication between the node and the gateway. The data represents ideal communication conditions under the assumption that the nodes are already connected to each other, there are no transmission errors during communication, and the interference by other technologies is omitted. For each technology, the required transmission time for a specific data amount is calculated based on the operation of the physical and medium access layer. Intelligence partitioning configurations result in varying data rate requirements, hence, for the transfer of large packets of data we consider several transmissions taking place continuously, until the required data amount is transferred, assuming that fragmentation at the network layer is possible. Furthermore, acknowledgements from the receiver are used to verify the reliability of data transmission according to protocol specifications. Equation (4) represents the calculation of the energy consumption for each transceiver analysed, based on the duration *t* and power consumption *P* of four operational states tx (transmitter), rx (receiver), idle, and sleep, to transfer the data *d*.

(4) $$E_{C\left(tech\right)}\left(d\right)=t_{tx}\left(d_{rx}\right)P_{tx}+t_{rx}\left(d_{tx}\right)P_{rx}+t_{idle}P_{idle}+t_{sleep}P_{sleep}$$

In addition to the wireless communication technologies used for the node to fog communication, we also need to include the latency for node to cloud or fog to cloud communication in our analysis, which would rely on the Internet. We measured the communication latency for the different data rate requirements produced by the configurations of intelligence partitioning in the three scenarios. For each set we recorded the latency for transfers done every minute to the cloud for a week. The final latency for each dataset is the average of the 90% most relevant latency information.

### 3.3. People Counting

The analysis of the node energy consumption alongside the WVSN latency is supported by data provided from the implementation of a people counting scenario, as shown in Table 2. It consists of a smart camera deployed in the city centre of Härnosand, Sweden to detect and count the people presence continuously throughout the day and night cycle. The information produced regarding the occupancy level of the city centre provides an important input for city planning in terms of business development and security. In addition, to comply with regulations regarding privacy when it comes to recording in public places, we used a low resolution thermal camera. The thermal sensor in FLIR Lepton 3 camera detects wavelengths in the range of 8 to 14 μm with a frame rate of 9 frames per second, 60 × 149 pixels, with 8 bits pixel depth. This resolution enables the detection of people, without features that can be used to reconstruct a person’s identity.

The people counting scenario relies on a set of image processing tasks starting with background modelling and subtraction as described in [31]. After subtraction we obtain the foreground image, a greyscale frame, which is later segmented based on a global predefined threshold. The segmented frame has a reduced size compared to the initial greyscale image, with 1 bit per pixel representation. To further improve the accuracy of the foreground detection, we apply morphological operations as erosion and dilation to the binary frame, removing the noise around the object of interest. The resulting binary image is used to detect and count people based on bounding box and Kalman filter methods. As we consider partitioning these image processing tasks between the computational elements present in the WVSN, we transfer frames to the fog and cloud. To reduce the communication bandwidth requirements, we use PNG compression for greyscale images, and CCITT group 4 compression for binary image compression. When the partitioning point is between the node and the fog/cloud precedes segmentation, we apply greyscale compression to each frame, otherwise we rely on binary image compression. Furthermore, we omit video compression methods from our analysis, because their artefacts from spatial and temporal compression would require adaption and verification of the image processing systems taken from literature. Hence, we have limited the design exploration to use only lossless image compression in order to fit the selected design cases.

### 3.4. Particle and Pedestrian Detection

In our analysis of the effects of intelligence partitioning in the node energy consumption and the overall latency, we have also included two scenarios of particle and pedestrian detection. The set of image processing tasks and the resulting data rate requirements based on the intelligence partitioning configurations vary between the two. The former scenario is developed by Imran et al. [32] and as shown in Table 3, it results in ten processing configurations with data rates varying from 259 bytes to 256,000 bytes, based on image processing tasks such as subtraction, segmentation, morphology, ROI and compression. The latter scenario designed by Maggiani et al. [33] provides and embedded architecture for pedestrian detection, relying on tasks such as gradient calculation, histogram of gradient, normalisation and SVM (Support Vector Machine). For our analysis we consider ten intelligence partitioning configurations, with the resulting data rates in the range from 11,264 bytes to 964,608 bytes, as shown in Table 4.

## 4. Results

The performance of a WVSN is strongly related to the fulfilment of constraints regarding the smart camera node energy consumption and the overall latency of both processing and communication of data between the processing layers. In this section we present the results of the node energy consumption and latency from applying intelligence partitioning to the three scenarios analysed. The latency and energy consumption calculations are done on frame-based image processing.

### 4.1. People Counting

**Figure 2:**
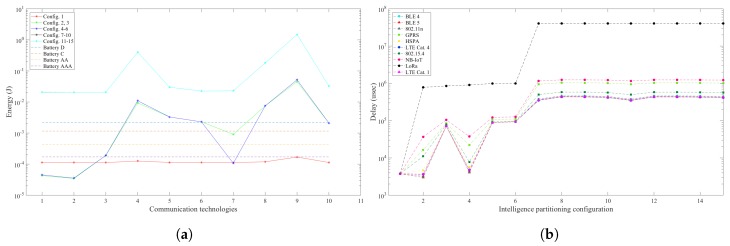
Total energy consumption and latency for the people counting scenario. (**a**) Energy consumption in the smart camera node and battery energy allowance per sample; (**b**) Latency of processing and communication in WVSN for each intelligence partitioning configuration.

For the people counting scenario, in Figure 2, there are the plots regarding the node energy consumption and the latency of the intelligence partitioning configurations considered. Configurations 11–15 have all the processing tasks allocated among the fog and the cloud layer, while the smart camera node only captures the frames, compresses and transfers them via wireless communication to the gateway. Configurations 7–10 only have background modelling and subtraction implemented in the smart camera, while the remaining tasks are computed remotely. A common factor among these configurations is the communication workload, where they are required to transfer greyscale frames of 8940 bytes, for each captured frame, to be further processed in the fog and/or cloud. Both the node energy consumption and the latency are at their peak for these configurations with about 2 × 10${}^{-2}$ Joules per frame, and a latency of 0.4 s.

Other configurations of interest are 1, 11 and 15, where all the processing is allocated in only one of the computational layers (the node, the fog and the cloud, respectively). This approach is common in many WVSN systems, as well as other IoT systems, facilitating the design process by concentrating the optimisation efforts to only one computational element. The implementation of all the tasks in the smart camera node delivers results within our expectations, with sub-optimal energy consumption 3 times higher than the energy consumption of the optimal intelligence partitioning configuration, while meeting the latency constraints. Configurations 11 and 15 have a worse performance with 3 orders of magnitude higher energy consumption, and latency beyond the real-time performance constraints.

Configurations 5, 8 and 9 rely on the use of all three computational layers for the image processing pipeline. As mentioned above, configurations 8 and 9 have a sub-optimal performance due to extensive data rate requirements. However, configuration 5 provides an optimal energy consumption for the smart camera node with 3.5 × 10${}^{-5}$ Joules per frame, while meeting the latency constraint with a latency of 8 × 10${}^{-2}$ s. As the latency is very close to the constraint of 0.1 s, the choice of wireless communication technology is of major importance in the preservation of the constraint. In this case, technologies such as LoRa and NB-IoT cannot be used due to high communication latency, while BLE 5 and 802.11n provide better performance.

The overall optimal intelligence partitioning configuration is configuration 2. It relies on the execution of background modelling, subtraction, segmentation and morphology in the smart camera, while detection and tracking are allocated in the fog, with the communication supported by BLE 5. This configuration provides the lowest energy consumption in the node with 3.4 × 10${}^{-5}$ Joules per frame, and the overall latency is 3 × 10${}^{-3}$ s, within the constraints of real-time performance.

### 4.2. Particle and Pedestrian Detection

**Figure 3:**
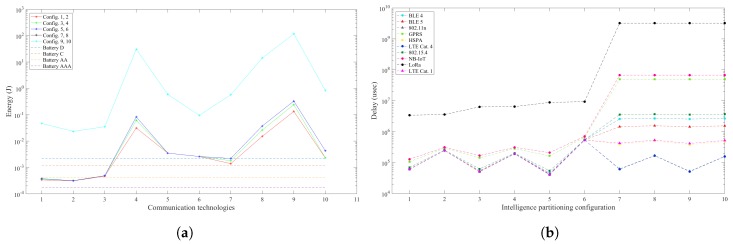
Total energy consumption and latency for the particle detection scenario. (**a**) Energy consumption in the smart camera node and battery energy allowance per sample; (**b**) Latency of processing and communication in WVSN for each intelligence partitioning configuration.

**Figure 4:**
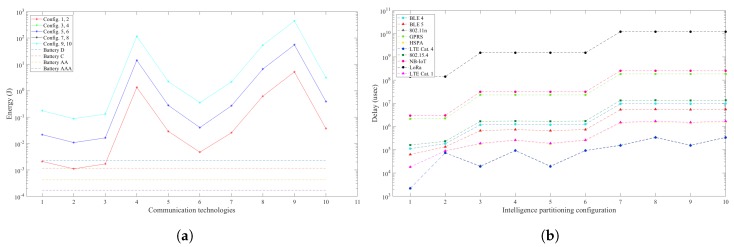
Total energy consumption and latency for the pedestrian detection scenario. (**a**) Energy consumption in the smart camera node and battery energy allowance per sample; (**b**) Latency of processing and communication in WVSN for each intelligence partitioning configuration.

The intelligence partitioning analysis omits the processing latency in the fog and cloud computing elements for the particle and pedestrian detection scenarios. Instead, it considers the communication latency for the required data rates. Figure 3 and Figure 4 show the results of the energy consumption and latency for the partitioning configurations considered in the particle detection and pedestrian detection scenarios. Configurations from 1 to 6 in Figure 3 have a comparable energy consumption of $3.1\;\times \;10^{-4}$ Joules, varying among them with approximately 1%, while the minimum latency is obtained in configuration 5 with $4\;\times \;10^{-2}$ s. Configurations 7 to 10 have a higher energy consumption due to higher communication workload with data rates of 250 kB per frame. The latency of all the configurations is above the 3.3 × 10${}^{-2}$ s threshold, showing that this system cannot deliver real-time performance regardless of intelligence partitioning configurations.

The pedestrian detection scenario, unlike the previous scenarios shows that the optimal partitioning configuration consists of implementing all the tasks considered in the smart camera node. This is due to the significant data reduction from 964,608 bytes to 11,264 bytes per frame from the image processing tasks, subsequently reducing the communication requirements from the node to the gateway. These results put an emphasis on the relationship between processing and the reduction in data rate requirements and the significant impact it can have on the efficiency of design space exploration.

## 5. Discussion

In this paper, we analyse how intelligence partitioning of the computational tasks between the smart camera node, the fog layer and the cloud affects the performance of the node in terms of energy consumption, while also considering real-time performance and coverage area for communication. The aim is to show how sensitive and therefore flexible the design exploration method needs to be, to optimise the performance of the node in an environment with node, fog and cloud computing. While traditional task partitioning in WVSN mainly consists of either in-node or remote processing, with intelligence partitioning, we prove that such an approach is not necessarily optimal, especially if we consider many constraints such as communication range, real-time performance and battery lifetime. The results obtained from the people counting, particle, and pedestrian detection scenarios suggest that intelligence partitioning can be used to enhance the performance of a WVSN. However, there are several elements that need to be considered related to processing and communication aspects.

**Figure 5:**
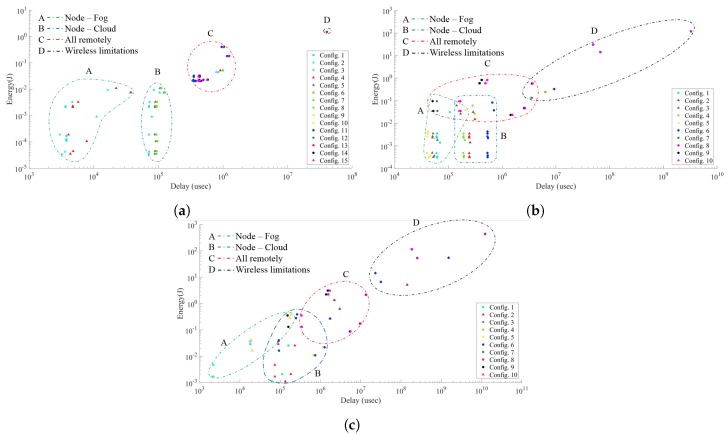
Delay and energy for intelligence partitioning configurations. (**a**) People counting scenario; (**b**) Particle detection scenario; (**c**) Pedestrian detection scenario.

In the results section, we showed that for the pedestrian detection scenario, the extensive processing provides marginal reduction in data rate requirements until the final processing task. Hence, in-node allocation of the image processing tasks proved to be more efficient. Instead, for the people counting and the particle detection scenarios there is a continuous data reduction resulting from the image processing tasks. Therefore, the energy efficient configurations consisted of distributed processing among the computational entities. To provide an insight at the inter-effects of processing and data rate requirements in the overall performance of the smart camera node in the WVSN, we plotted the latency against the node energy consumption for each of the scenarios as shown in Figure 5. For the people counting scenario, we have data rates from 4 to 3179 Bytes per frame, with Figure 5a showing a clear distinction between the intelligence partitioning configurations where the tasks are allocated between node-fog, node-cloud and all remotely in either the fog or the cloud. The performance advantages of intelligence partitioning are visible, with the node-fog partitioning configuration providing the highest efficiency in terms of energy consumption and latency.

The particle detection scenario has higher data rate requirements, reaching a range of 259 to 256,000 Bytes per frame. This results in communication limitations for IoT technologies such as LoRa and NB-IoT. The increment in communication workload diminishes the distinction between the intelligence partitioning configurations, with the efficiency trade off marked mainly by communication technologies as shown in Figure 5b. The data rate requirements in the pedestrian detection scenario vary from 11,264 to 964,608 Bytes, and are the highest among the scenarios considered. Subsequently, in Figure 5c the latency and energy consumption of the wireless communication technology defines the optimal intelligence partitioning configuration. These results emphasise the importance of processing and communication inter-effects in design space exploration.

In the motivation of intelligence partitioning analysis, we mention scenarios such as environmental monitoring and smart city, alongside their prospective need for a network of battery-operated smart cameras to cover large areas and perform a multitude of tasks within a satisfactory battery lifetime. Considering this, one key aspect in selecting the optimal partitioning configuration and communication technology is not only the node energy consumption, but also the communication range. For all the scenarios reviewed above, the overall optimal energy consumption is provided by configurations relying on communication technologies with a low communication range of about 100 m (BLE, 802.11n, 802.15.4). A strong constraint regarding the communication range would shift the optimal partitions to configurations with LTE Cat.1 and Cat.4 technologies, resulting in higher energy consumption in the range of 3 to 7 times higher than the overall optimum.

Both people counting and particle detection had their best performance in terms of energy consumption and latency for configurations where the tasks are partitioned between the computational layers. Nevertheless, if we consider both systems with hard real-time performance, only the people counting scenario can be implemented. For the particle detection scenario, the processing latency is high enough that the addition of the wireless communication latency for data transfers to the fog makes it impossible to meet real-time performance constraints. Furthermore, if we continue under the assumption of hard real-time constraints, the use of cloud computing can result in an unstable system, caused by the high variation of communication time through the Internet. We analysed the latency for node/fog to cloud communication for different data rate requirements, and the results showed no correlation between the communication workload in terms of frame size, and the latency. Hence, for systems without stringent real-time constraints, the aggregation of data in the gateway and allocation of intensive computational tasks in the cloud could have a positive impact on the camera node energy efficiency. However, this does not mean that we can simply stream all the data collected from the camera and process it in the cloud. The energy consumption of all three scenarios showed that fully remote processing is inefficient for the smart camera node energy consumption due to the high communication workload for the node to gateway communication.

**Figure 6:**
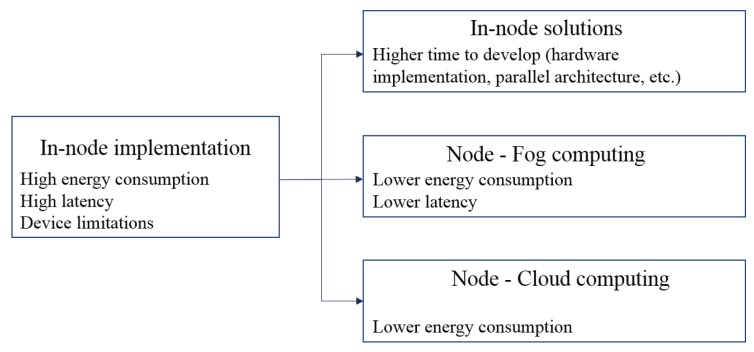
Representation of the computational shift inspired by intelligence partitioning, and the expected effects.

Figure 6 summarises the observations resulting from intelligence partitioning. For architectures where in-node implementation of the computational tasks results in high energy consumption and high latency, we observe three prominent solutions. In-node optimisation through fine-tuned hardware implementation of computational blocks that can be demanding in processing time, can improve both energy consumption and latency. However, such solutions require a long time to be developed and considerations about the embedded device capacity. A less time consuming option can be to shift the architecture towards a node-fog computing system, which, depending on the application requirements, can meet real-time performance requirements. Furthermore, if the system has no constraints regarding latency, cloud computing can be an optimal solution, reducing the node energy consumption while using extensive computational and memory resources at a low cost.

## 6. Conclusions

This paper proposes intelligence partitioning as an approach towards energy efficient battery-operated smart cameras. The data obtained from the analysis of three implementation scenarios disproves the general assumption that the allocation of all the computational tasks either in-node or remotely is the most energy efficient approach. It also shows that there is no golden intelligence partitioning point, but there are different regions where performance improves depending on the definition and hierarchy of constraints such as energy consumption, latency, or communication range. The overview of latency and energy consumption underlined the importance of the communication workload in the energy efficiency of the node. Understanding the relationship between additional processing and the resulting reduction in communication data rates is a key element in defining if intelligence partitioning can enhance the given system. Considering the requirement for small form factor in IoT devices, intelligence partitioning enables the implementation of demanding computer vision applications within satisfactory battery lifetime thresholds.

The problem of the distribution of the computational tasks for the node energy efficiency is greater than could be presented in this paper. Another important consideration that will need to be included as we move forward with WVSN architectures is security. The distribution of the computational tasks and data results in a more exposed system, hence, the inclusion of encryption algorithms in the smart camera node will become a strong requirement in future WVSN, subsequently affecting design space exploration.

## Figures and Tables

**Table 1:** Configurations of communication technologies.

Category	Technology	Plotting Order
LAN		
	BLE 4.2 [20]	(1)
	BLE 5 [21]	(2)
	802.11n [22]	(3)
Cellular		
	GPRS [23]	(4)
	HSPA [24]	(5)
	LTE Cat. 4 [25]	(6)
IoT		
	802.15.4 [26]	(7)
	NB-IoT [27]	(8)
	LoRa [28]	(9)
	LTE Cat. 1 [29]	(10)

**Table 2:** Intelligence partitioning configurations in accordance with Equation (1) for the people counting scenario. (SC - smart camera, and Cl - cloud)

Conf.	Background Modelling	Segmentation	Morphology	Detection & Tracking	Data Rate
1	SC	SC	SC	SC	4
2	SC	SC	SC	Fog	75
3	SC	SC	SC	Cl	75
4	SC	SC	Fog	Fog	91
5	SC	SC	Fog	Cl	91
6	SC	SC	Cl	Cl	91
7	SC	Fog	Fog	Fog	3179
8	SC	Fog	Fog	Cl	3179
9	SC	Fog	Cl	Cl	3179
10	SC	Cl	Cl	Cl	3179
11	Fog	Fog	Fog	Fog	3179
12	Fog	Fog	Fog	Cl	3179
13	Fog	Fog	Cl	Cl	3179
14	Fog	Cl	Cl	Cl	3179
15	Cl	Cl	Cl	Cl	3179

**Table 3:** Intelligence partitioning configurations in accordance with Equation (1) for the particle detection scenario. (SC - smart camera, and Cl - cloud)

Conf.	Image Capturing	Background Modelling	Segmentation	Morphology	ROI	Compression	Other	Data Rate
1	SC	SC	SC	SC	SC	SC	Fog	259
2	SC	SC	SC	SC	SC	SC	Cl	259
3	SC	SC	SC	SC	Fog	SC	Fog	500
4	SC	SC	SC	SC	Cl	SC	Cl	500
5	SC	SC	SC	Fog	Fog	SC	Fog	680
6	SC	SC	SC	Cl	Cl	SC	Cl	680
7	SC	SC	Fog	Fog	Fog	-	Fog	256,000
8	SC	SC	Cl	Cl	Cl	-	Cl	256,000
9	SC	Fog	Fog	Fog	Fog	-	Fog	256,000
10	SC	Cl	Cl	Cl	Cl	-	Cl	256,000

**Table 4:** Intelligence partitioning configurations in accordance with Equation (1) for the pedestrian detection scenario. (SC - smart camera, and Cl - cloud)

Conf.	Image Capturing	Gradient	Histogram	Normalisation	SVM	Other	Data Rate
1	SC	SC	SC	SC	SC	Fog	11,264
2	SC	SC	SC	SC	SC	Cl	11,264
3	SC	SC	SC	SC	Fog	Fog	119,808
4	SC	SC	SC	SC	Cl	Cl	119,808
5	SC	SC	SC	Fog	Fog	Fog	119,808
6	SC	SC	SC	Cl	Cl	Cl	119,808
7	SC	SC	Fog	Fog	Fog	Fog	964,608
8	SC	SC	Cl	Cl	Cl	Cl	964,608
9	SC	Fog	Fog	Fog	Fog	Fog	964,608
10	SC	Cl	Cl	Cl	Cl	Cl	964,608
